# Chromosome spreading of the (TTAGGG)n repeats in the *Pipa carvalhoi* Miranda-Ribeiro, 1937 (Pipidae, Anura) karyotype

**DOI:** 10.3897/CompCytogen.v13i3.35524

**Published:** 2019-10-14

**Authors:** Michelle Louise Zattera, Luana Lima, Iraine Duarte, Deborah Yasmin de Sousa, Olívia Gabriela dos Santos Araújo, Thiago Gazoni, Tamí Mott, Shirlei Maria Recco-Pimentel, Daniel Pacheco Bruschi

**Affiliations:** 1 Programa de Pós-Graduação em Genética, Departamento de Genética, Universidade Federal do Paraná (UFPR), Centro Politécnico, Jardim das Américas, 81531-990,Curitiba, Paraná State, Brazil Universidade Federal do Paraná Curitiba Brazil; 2 Instituto de Ciências Biológicas e da Saúde, Universidade Federal do Alagoas (UFAL), Avenida Louriva Melo Mota S/N, 57072-900, Maceió, Alagoas State, Brazil Universidade Federal do Alagoas Maceió Brazil; 3 Departamento de Biologia, Instituto de Biociências, Universidade Estadual Paulista (Unesp) – Câmpus Rio Claro, Avenida 24 A 1515, Bela Vista, 13506-900, Rio Claro, São Paulo State, Brazil Universidade Estadual Paulista Rio Claro Brazil; 4 Instituto de Biologia, Departamento de Biologia Estrutural e Funcional, Universidade Estadual de Campinas (UNICAMP), Avenida Bertrand Russel S/N, Barão Geraldo, 13083-865, Campinas, São Paulo State, Brazil Universidade Estadual de Campinas Campinas Brazil

**Keywords:** Pipidae, chromosome banding, interstitial telomeric sequences, rearrangements, chromosome evolution

## Abstract

Pipidae is a clade of Anura that diverged relatively early from other frogs in the phylogeny of the group. Pipids have a unique combination of morphological features, some of which appear to represent a mix of adaptations to aquatic life and plesiomorphic characters of Anura. The present study describes the karyotype of *Pipa carvalhoi* Miranda-Ribeiro, 1937, including morphology, heterochromatin distribution, and location of the NOR site. The diploid number of *P. carvalhoi* is 2n=20, including three metacentric pairs (1, 4, 8), two submetacentric (2 and 7), three subtelocentric (3, 5, 6), and two telocentric pairs (9 and 10). C-banding detected centromeric blocks of heterochromatin in all chromosome pairs and the NOR detected in chromosome pair 9, as confirmed by FISH using the rDNA 28S probe. The telomeric probes indicated the presence of interstitial telomeric sequences (ITSs), primarily in the centromeric region of the chromosomes, frequently associated with heterochromatin, suggesting that these repeats are a significant component of this region. The findings of the present study provide important insights for the understanding of the mechanisms of chromosomal evolution in the genus *Pipa*, and the diversification of the Pipidae as a whole.

## Introduction

Chromosome studies provide important insights into the diversification of karyotypes and represent an effective approach for the identification of homologies among species (Targueta et al. 2018). This approach provides a systematic understanding of the rearrangements of the genome that have occurred during the evolutionary history of the target group.

Pipids are a clade of anurans that diverged relatively early from other frogs in the phylogeny of the group (Pyron and Wiens 2011). Pipids have a unique combination of morphological features, some of which appear to represent a mix of adaptations to aquatic life and plesiomorphic characters of Anura (Cannatella and Trueb 1988, Cannatella 2015, Araújo et al. 2017). The frogs of the family Pipidae dwell in freshwater environments and have behavioral and physiological features that are unique in anuran amphibians, making this group an excellent model for evolutionary studies (Cannatella and Trueb 1988, Cannatella and De Sá 1993, Pough et al. 2001). The family currently includes four genera: *Hymenochirus* Boulenger, 1896 (4 species), *Pseudohymenochirus* Chabanaud, 1920 (1 species), *Xenopus* Wagler, 1827 (29 species), and *Pipa* Laurenti, 1768 (7 species), which are distributed in sub-Saharan Africa and South America (Frost 2019).

However, based on molecular phylogenetic inferences and presumed ancestral diploid numbers, some authors have distinguished a fifth lineage, *Silurana*, which includes all the species derived from an ancestor with 2n = 20 (Evans et al. 2004, Pyron and Wiens 2011), from *Xenopus*, which has an ancestral diploid number of 2n = 18. Evans et al. (2015) suggested that *Xenopus* should be divided into two subgenera, *Xenopus* and *Silurana*. Other authors consider *Xenopus* and *Silurana* a monophyletic clade, without the necessity of separation of subfamilies or genera between them (e.g., De Sá and Hillis 1990, Cannatella and De Sá 1993, Graf et al. 1996), in this work we will consider them as a single group, *Xenopus tropicalis* group (Frost 2019).

*Pipa* is the only non-African representative of the Pipidae, and evidences from a number of different sources indicates that this South American lineage is derived from an ancestor closely related to the extant members of the genus *Hymenochirus*. Pipidae was widely distributed in Gondwana and after its splintering, those lineages had distributions associated with the Afro-Tropical (*Hymenochirus*, *Pseudhymenochirus* and *Xenopus*) and Neotropical Regions (*Pipa*). The historical isolation resulted in the diversification of the ancestral lineage of the genus *Pipa*, which is found in South America, as far north as Panama (Trueb et al. 2005, Frost 2019).

The genus currently contains seven species: *P. arrabali* Izecksohn, 1976, *P. aspera* Mueller, 1924, *P. carvalhoi* Miranda-Ribeiro, 1937, *P. myersi* Trueb, 1984, *P. parva* Ruthven & Gaige, 1923, *P. pipa* (Linnaeus, 1758), and *P. snethlageae* Muller, 1914 (Frost 2019). In most cases, the only cytogenetic information available for the pipid species is the diploid number. The *Xenopus* + *Silurana* lineages (*sensu*Evans et al. 2015), the sister group of *Pipa*, have the largest number of karyotyped species, including recurrent cases of polyploidy, with the chromosomal number being used as a criterion for the description of new species (Evans et al. 2015). The karyotypes of *Hymenochirus boettgeri* (Tornier, 1896) and *Pseudohymenochirus merlini* Chabanaud, 1920 (a monotypic genus) were described recently, filling gaps in the chromosomal history of the Pipidae (Mezzasalma et al. 2015). In the case of *Pipa* the available cytogenetic data are limited to the diploid numbers for *P. parva* (2n = 30) and *P. pipa*, with 2n = 22 (Wickbom 1950, Morescalchi 1968, Morescalchi et al. 1970). *Pipa carvalhoi* present a diploid number of 20 chromosomes; however, this information was only determined based on an ideogram published by Mezzasalma et al. (2015), which was inferred based on the data of an unpublished degree thesis (Pfeuffer-Friederich 1980).

As no data whatsoever are available for the other five *Pipa* species, further studies will be essential for the understanding of the genomic rearrangements that have occurred during the adaptive radiation of this lineage in South America. Here, we describe the karyotype of *P. carvalhoi*, including the position of the NORs and the distribution pattern of the heterochromatin. We also documented the intrachromosomal spread of the telomeric (TTAGGG)n motifs and discuss these findings in the context of the phylogenetic scenario of the family Pipidae.

## Material and methods

### Samples

We analyzed three specimens of *Pipa carvalhoi* collected in Buerarema (three male), Bahia state, Brazil, and three from Buíque (two male + one juvenile), Pernambuco state, Brazil. The collection of specimens was authorized by SISBIO/Instituto Chico Mendes de Conservação da Biodiversidade through protocol number 55481-1. The specimens were deposited in the “Célio Fernando Baptista Haddad” Amphibian Collection (CFBH), on the Rio Claro campus of São Paulo State University (UNESP) and in Natural History Museum in Universidade Federal de Alagoas (MHN-UFAL).

### Staining procedures

The chromosomal preparations were obtained from intestinal and testicular cells treated with 2% colchicine for 4 hours, using techniques modified from King and Rofe (1976) and Schmid (1978). The mitotic metaphases were stained with 10% Giemsa for karyotyping. The heterochromatic regions were identified by C-banding, using the technique described by Sumner (1972) and C-banding + DAPI. We detected the NORs using the Ag-NOR method (Howell and Black 1980). The chromosomes were ranked and classified according to the scheme of Green and Sessions (1991).

### Fluorescence *in situ* hybridization

Loci of 28S rDNA were detected fluorescence *in situ* hybridization (FISH). We used the 28S fragment isolated by Bruschi et al. (2012) to detect the rDNA genes. This probe was PCR-labeled with digoxigenin and hybridized following the protocol of Viegas-Péquignot (1992). Finally, the vertebrate telomeric (TTAGGG)n sequence probe was obtained by PCR amplification and labeling, based on Ijdo et al. (1991).

## Results

**Figure 1.**
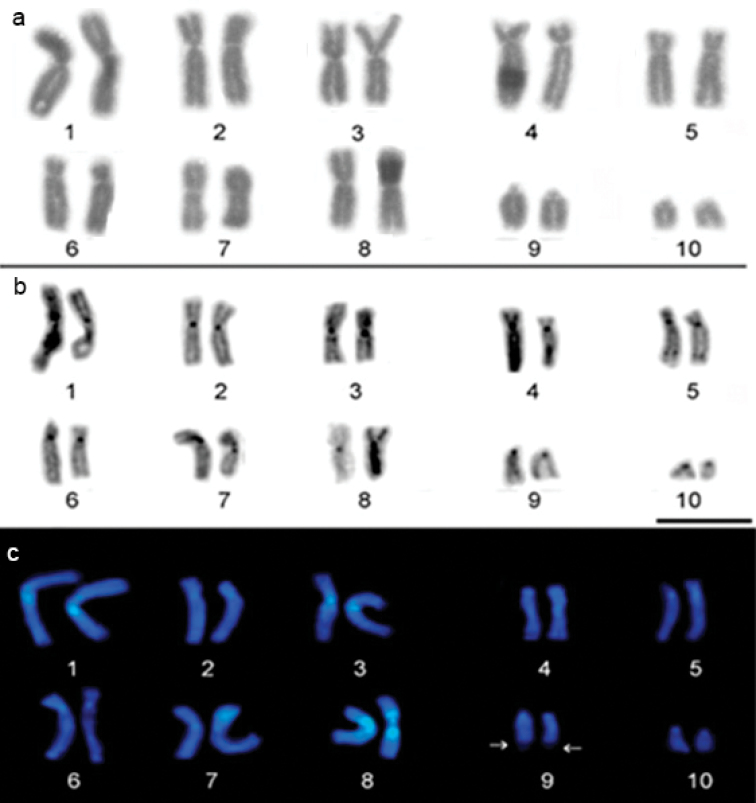
Karyotype of *P. carvalhoi*. **a** Prepared by conventional Giemsa staining **b** C-banding and **c** DAPI staining. The arrow indicates the NOR site.

The diploid number of the *P. carvalhoi* karyotype was 2n = 20 chromosomes (Fig. 1). The karyotype contains three metacentric pairs (1, 3, 8), two submetacentric (2 and 7), three subtelocentric (4, 5, 6), and two telocentric pairs, 9 and 10 (Fig. 1). The same karyotype was recorded in both populations.

The C-banding technique detected centromeric blocks of heterochromatin in all chromosome pairs. Interstitial heterochromatin blocks were also detected in the long arms of pair 5 (Fig. 1B). Pericentromeric C-positive banding was observed in the long arm of pair 3, and in the short arm of the submetacentric pair 7 (Fig. 1B). The centromeric blocks of heterochromatin presented DAPI-positive signals in all the chromosomes, in addition to pericentromeric heterochromatin in pair 3 (Fig. 1C). The DAPI staining also revealed a conspicuous bright signal in the pericentromeric regions of both arms of pair 3 and 8. Neither of these features were revealed by the C-banding (Fig. 1C).

**Figure 2.**
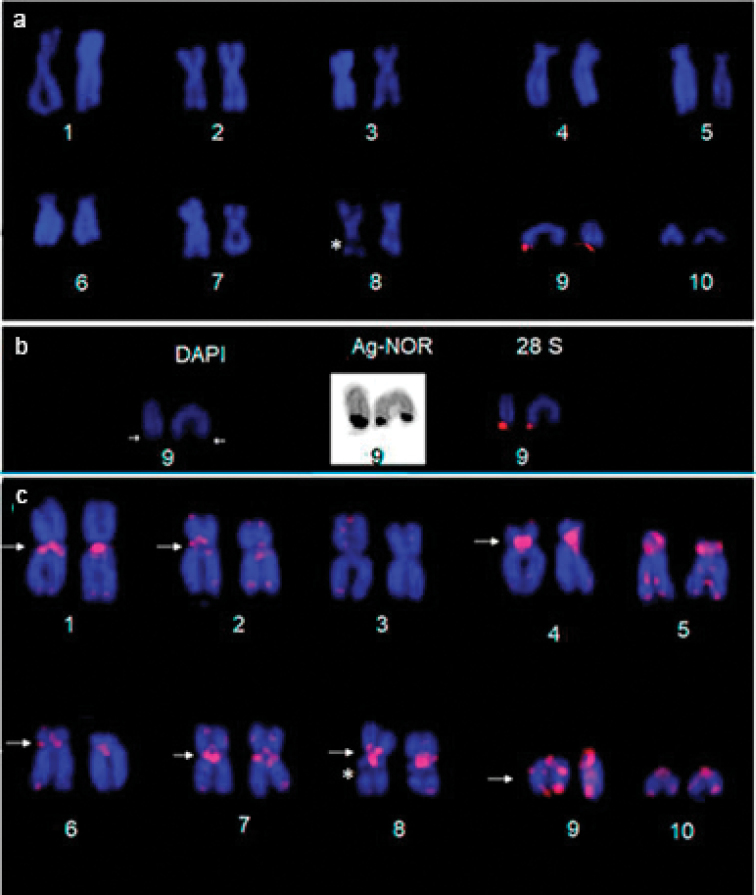
Fluorescence *in situ* hybridization in *P. carvalhoi* karyotype. **a** Hybridized with the 28S rDNA probe **b** the NOR-bearing chromosome highlighted by DAPI-staining, the Ag-NOR method, and FISH with 28S rDNA **c***In situ* hybridization with the telomeric probe in the karyotype of *P. carvalhoi* from Pernambuco, Brazil. The arrows in **c** indicate the interstitial telomeric sequences (ITSs) and the constriction in chromosome 8 are indicates by asterisk.

Under conventional Giemsa staining, a secondary constriction was observed in the subterminal regions of the homologs of pair 9, which coincides with the NOR site (in both populations), detected by the Ag-NOR method and confirmed by FISH using the rDNA 28S probe (Fig. 2A), and this region was DAPI-negative (Fig. 2B). The telomeric probe hybridized all the telomeres in the chromosomes of *P. carvalhoi*. Conspicuous signals of Interstitial Telomeric Sequences (ITSs) can be observed in the centromeric/pericentromeric region of the homologs of pairs 1, 2, 4, 5, 6, 7, and 8, and in the interstitial region of the long arm of chromosome pair 9. A secondary constriction was also observed in chromosome pair 8 (Fig. 2A, C).

## Discussion

The chromosomal evolution of the pipids appears to have involved complex rearrangements, including recurrent polyploidization events and associated shifts in the diploid number (Table 1). In the present study, we redescribed the karyotype of *P. carvalhoi*, including the distribution of the heterochromatin and the NOR site. In the phylogenetic reconstructions of the superfamily Pipoidea proposed by Pyron and Wiens (2011) and Irisarri (2011), the 2n = 22 diploid number was identified as the plesiomorphic condition, based on the karyotype of *Rhinophrynus dorsalis* Duméril & Bibron, 1841 (Bogart and Nelson 1976), the only member of the Rhinophrynidae.

**Table 1.** Detailed cytogenetic data available for species of the Pipidae family. NOR: Nucleolar Organizer Region; M= metacentric; SM= submetacentric; ST=subtelocentric; T=telocentric; p= short arm; q=long arm; (–) no data.

Species	Ploidy level	Karyotype formula	NOR site	Reference
***Xenopus tropicalis* group**				
*X. tropicalis*	2n = 20	2 M + 14 SM + 4 A	5q	Tymowska and Fischberg 1973; Uehara et al. 2002
*X. epitropicalis*	4n = 40	4M + 28 SM+ 8 A	5q	Tymowska and Fischberg 1982; Tymowska 1991
*X.* new tetraploid 1	4n = 40	4M + 28 SM+ 8 A	5q	Tymowska 1991; Evans et al. 2004
*X.* new tetraploid 2	4n = 40	4M + 28 SM+ 8 A	5q	Evans et al. 2004
***Xenopus laevis* group**				
*X. borealis*	4n = 36	6 M+ 14 SM+ 2 ST + 14 T	4p	Tymowska and Fischberg 1973; Tymowska 1991
*X. clivii*	4n = 36	6 M+ 14 SM+ 2 ST + 14 T	4p	Tymowska and Fischberg 1973; Tymowska 1991
*X. fraseri*	4n = 36	6 M+ 14 SM+ 2 ST + 14 T	6q	Tymowska and Fischberg 1973; Tymowska 1991
*X. gilli*	4n = 36	6 M+ 14 SM+ 2 ST + 14 T	12p	Tymowska and Fischberg 1973; Tymowska 1991
*X. laevis laevis*	4n = 36	6 M+ 14 SM+ 2 ST + 14 T	12p	Tymowska 1991
*X. laevis bunyoniensis*	4n = 36	6 M+ 14 SM+ 2 ST + 14 T	–	Tymowska 1991
*X. laevis petersi*	4n = 36	6 M+ 14 SM+ 2 ST + 14 T	–	Tymowska and Fischberg 1973; Tymowska 1991
*X. laevis poweri*	4n = 36	6 M+ 14 SM+ 2 ST + 14 T	–	Tymowska 1991
*X. laevis sudanensis*	4n = 36	6 M+ 14 SM+ 2 ST + 14 T	–	Tymowska 1991
*X. laevis victorianus*	4n = 36	6 M+ 14 SM+ 2 ST + 14 T	–	Tymowska and Fischberg 1973; Tymowska 1991
*X. largeni*	4n = 36	6 M+ 14 SM+ 2 ST + 14 T	–	Tymowska 1991
*X. muelleri*	4n = 36	6 M+ 14 SM+ 2 ST + 14 T	4p	Tymowska and Fischberg 1973; Tymowska 1991
*X. pygmaeus*	4n = 36	6 M+ 14 SM+ 2 ST + 14 T	6q	Loumont 1986
*X.* sp. nov. VI	4n = 36	6 M+ 14 SM+ 2 ST + 14 T	4p	Tymowska 1991
*X.* sp. nov. IX	4n = 36	6 M+ 14 SM+ 2 ST + 14 T	12p	Tymowska 1991
*X. amieti*	8n = 72	12 M + 28 SM + 4 ST + 28 T	5q	Kobel et al. 1980
*X. andrei*	8n = 72	12 M + 28 SM + 4 ST + 28 T	18q	Loumont 1983
*X. boumbaensis*	8n = 72	12 M + 28 SM + 4 ST + 28 T	6p+ 4p	Loumont 1983
*X. itombwensis*	8n = 72	12 M + 28 SM + 4 ST + 28 T	–	Evans et al. 2008
*X. lenduensis*	8n = 72	12 M + 28 SM + 4 ST + 28 T	–	Evans et al. 2011
*X. vestitus*	8n = 72	12 M + 28 SM + 4 ST + 28 T	12p	Tymowska 1991
*X. wittei*	8n = 72	12 M + 28 SM + 4 ST + 28 T	12p	Tymowska 1991
*X.* sp. nov. X	8n = 72	12 M + 28 SM + 4 ST + 28 T	18q	Tymowska 1991
*X. longipes*	12n = 108	18 M + 42 SM + 6 ST + 42 T	7p	Loumont and Kobel 1991
*X. ruwenzoriensis*	12n = 108	18 M + 42 SM + 6 ST + 42 T	11q	Tymowska and Fischberg 1973; Tymowska 1991
X. cf. boumbaensis	12n = 108	18 M + 42 SM + 6 ST + 42 T	7p	Evans 2007
*X.* sp. nov. VIIIa	12n = 108	18 M + 42 SM + 6 ST + 42 T	7p	Tymowska 1991
**Genus *Pseudhymenochirus***				
*P. merlini*	2n = 20	8 M + 4 SM + 6 ST + 2 T	10q	Mezzasalma et al. 2015
**Genus *Hymenochirus***				
*H. boettgeri*	2n = 20	14 M + 2 SM + 4 ST	4p	Mezzasalma et al. 2015
**Genus *Pipa***				
*P. carvalhoi*	2n = 20	6 M+ 4 SM+6 ST + 4 T	9q	Present study
		8 M + 8 SM + 4 T^†^		Mezzasalma et al. 2015
*P. pipa*	2n = 22	8 M + 14 A	–	Wickbom 1950
		6M + 2ST + 14A		Morescalchi et al. 1970
*P. parva*	2n = 30	30 T	–	Morescalchi 1981

^†^Chromosomal formula shown by Mezzasalma et al. 2015 was based in Pfeuffer-Friederich 1980 and Sachsse 1980.

Subsequently, Mezzasalma et al. (2015) proposed that the ancestral karyotype of Pipidae had a diploid number of 2n = 20, based on the conserved diploid numbers observed in *Xenopus* (= *Silurana*) *tropicalis* and *Hymenochirus boettgeri* + *Pseudhymenochirus merlini*. The phylogenetic inferences of Evans et al. (2004) and Irisarri (2011) indicated the existence of two clades in the clawed frogs (*Xenopus*), with a well-supported synapomorphy of the diploid number, which divides the species of this genus into two separate lineages: the subgenus Silurana (2n = 20) and the subgenus Xenopus, which has the primitive diploid number (2n = 18). The diploid number (2n = 20) found in *Xenopus* (= *Silurana*) *tropicalis* (Gray, 1864) and the polyploidy of the species derived from this form [2n = 4x = 40: *Xenopus* (= *Silurana*) *calcaratus* Peters, 1877; *Xenopus* (= *Silurana*) *epitropicalis* Fischberg, Colombelli and Picard 1982; *Xenopus* (= *Silurana*) *mellotropicalis*Evans et al. 2015] correspond to a retention of the plesiomorphic condition of the pipids (Mezzasalma et al. 2015).

The diploid number (2n = 20) recorded here in *P. carvalhoi* also corresponds to a retention of the plesiomorphic condition of the pipids, and an overview of all the known karyotypes of pipid species indicates that the morphology of pairs 1, 2, 3, and 4 is highly conserved, as it is in the outgroup, *Rhinophrynus dorsalis* (Mezzasalma et al. 2015). Despite the conservative karyology of the principal pipid clades, the known diploid numbers of *Pipa* species vary considerably. The two other species for which data are available are *P. parva*, which has a karyotype (2n = 30) composed entirely of telocentric pairs (Wickbom 1950, Morescalchi 1968), and *P. pipa*, which has a diploid number of 2n = 22 (Morescalchi et al. 1970).

The comparison of the karyotypes of *P. carvalhoi* and *P. pipa* (Wickbom 1950, Morescalchi et al. 1970) indicates interspecific chromosomal homologies of the metacentric and submetacentric pairs 1, 2, 3, and 4. The minor differences between the *P. pipa* karyotypes published by Wickbom (1950) and Morescalchi et al. (1970) are derived from variation in the chromosomal nomenclature adopted in the two studies, rather than any real karyotype differences among the *P. pipa* populations. As the *P. parva* karyotype contains only telocentric pairs, the recognition of chromosome homologies with other *Pipa* species are currently restricted by the lack of appropriate markers.

The pericentromeric heterochromatin block in the homologs of pair 3 of *P. carvalhoi* could be a common feature of pipid karyotypes. Interestingly, this heterochromatin block, is also present in *Xenopus* (= *Silurana*) *tropicalis* (Tymowska & Fischberg, 1982), *Hymenochirus boettgeri*, and *Pseudhymenochirus merlini* karyotype (Mezzasalma et al. 2015), which all have a diploid number of 2n = 20 chromosomes. As the configuration of the heterochromatin is a valuable marker for the interspecific comparison of karyotypes, the unique non-centromeric heterochromatin blocks found in some of the chromosomes of *P. carvalhoi* constitute an important diagnostic trait for the analysis of the interspecific variation in the pipids, based on C-banding.

We detected interstitial telomeric sequences (ITSs) in the centromeric/pericentromeric region of the metacentric and submetacentric chromosomes of the *P. carvalhoi* karyotype. Based in Mezzasalma et al. (2015) hypothesis, the *P. carvalhoi* karyotype have been diversification from primitive Pipidae karyotype mainly by pericentromeric inversion involved pairs 3, 6, 8-10. In our data, the pericentromeric ITS detected in homologues of pairs 6, 8 and 9 validated this hypothesis, highlights the role of the intrachromosomal rearrangements shaping karyotype diversification in Pipidae. The pericentromeric inversion involved pair 3 occurred without repositioned telomeric repeats that justify absence of the ITS in this metacentric pair. Canonical telomeric repeats are located in the terminal regions of the chromosomes, but several vertebrate species have blocks of (TTAGGG)n repeats in non-terminal regions of their chromosomes (Mayne et al. 1990, Bolzán 2017). Non-telomeric (TTAGGG)n repeats have been described frequently in anuran species see (Bruschi et al. 2014, Schmid and Steinlein 2016, Schmid et al. 2018). For example, Nanda et al. (2008) reported the presence of ITSs in pipid chromosomes for the first time, detecting a wealth of non-telomeric (TTAGG)n repeats in the chromosomes of *Xenopus clivii* Peracca, 1898, in pair 17 of the *Xenopus Laevis* (Daudin, 1802) karyotype, and associated with the NOR in *X. borealis* and *X. muelleri* (Peters, 1844). Interestingly, the ITS markers were fundamental to the discrimination of the karyotypes of these four species, which all share the same diploid number (2n = 36) and have highly uniform chromosome morphology, when analyzed using a classical cytogenetic approach. The ITSs distinguish *X. clivii*, which has much more numerous ITSs in comparison with the other *Xenopus* karyotypes.

Despite being an unusual feature of vertebrate genomes, we found ITS sites in euchromatic regions (in pair 9, for example), as found in some other anuran species. Schmid and Steinlein (2016) proposed ‘large ITSs in restricted euchromatic regions (restricted eu-ITSs)’ as a new category of ITS in anuran karyotypes. These euchromatic ITSs have already been documented in chromosome pairs 2 and 9 of *Hypsiboas boans* (Linnaeus, 1758) (Schmid and Steinlein 2016), which is consistent with the presence of these markers in pair 9 of *P. carvalhoi*.

Adopting the parsimony criterion, we rejected the hypothesis that the ITSs detected in the *P. carvalhoi* karyotype are remnants of centric (Robertsonian) fusions, given that *P. carvalhoi* has the plesiomorphic pipid diploid number. However, for some chromosomes (pairs 6, 8 and 9) theses ITS to confirm occurrence of the intrachromosomal rearrangements during evolution of this karyotype. Already, for others ITS signals, our data support the conclusion that the presence of the intrachromosomal telomeric motif (TTAGGG)n represents a component of the repetitive DNA sequences spread throughout these chromosomes. Furthermore, the ITSs found in the *P. carvalhoi* chromosomes coincide with the heterochromatic blocks detected by C-banding in chromosomes 1, 2, 4, 5 and 7. The role of telomeric repeats as repetitive motifs of part of the satellite DNA has already been described in a number of rodent genera, with a unique signal being found in the pericentromeric heterochromatin together with Msat-160 or in telomeric probes, in experiments with co-located het-ITSs and the Msat-160 satellite DNA (Rovatsos et al. 2011).

Ruiz-Herrera et al. (2008) proposed a model to account for the presence of short ITSs in the genome of vertebrates, in which the sequences originate from the insertion of telomeric repeats during the repair of double-strand breaks (DBSs) in the DNA, which may occur either with or without the intervention of telomerase, with the telomerase-mediated repair of the DSBs possibly leading to the appearance of ITSs. Bolzán and Bianchi (2006) concluded that the amplification of these sequences may be related to (i) the insertion of telomeric repeats during the repair of double-strand breaks or (ii) transposable elements. In the former case (i), the telomerase may catalyze the addition of telomeric sequences directly to non-terminal regions through the direct addition of (TTAGGG)n repeats to the ends of broken chromosomes (chromosome healing). The amplification of the ITSs may also occur through unequal crossing over between the repeats of sister chromatid breakage-fusion-bridge cycles, replication slippage (Lin and Yan 2008), gene conversion, and excision and reintegration events through the ‘rolling circle’ mechanism (Bolzán 2017).

## Conclusion

Overall, then, the results of the present study indicate that *P. carvalhoi* has a karyotype of 2n = 20 chromosomes, supporting that this chromosome formula represents the pleiomorphic condition of the pipids, with interspecific chromosomal homologies indicating a highly conserved karyotype configuration. The presence of ITSs in some chromosomes may have originated independently during the chromosomal evolution of this species which in others pairs correspond to evidences of the pericentromeric inversions occurred during Pipidae karyotype diversification. The findings of the present study provide important insights into the mechanisms of chromosomal evolution in the genus *Pipa* and the diversification of the family Pipidae as a whole.
